# Taking a Stand: The Effects of Standing Desks on Task Performance and Engagement

**DOI:** 10.3390/ijerph14080939

**Published:** 2017-08-21

**Authors:** Laura E. Finch, A. Janet Tomiyama, Andrew Ward

**Affiliations:** 1 Department of Psychology, University of California, Los Angeles, CA 90095, USA; tomiyama@psych.ucla.edu; 2 Department of Psychology, Swarthmore College, Swarthmore, PA 19081, USA; award1@swarthmore.edu

**Keywords:** standing desk, sit-stand workstation, performance, reading comprehension, creativity, engagement

## Abstract

Time spent sitting is associated with negative health outcomes, motivating some individuals to adopt standing desk workstations. This study represents the first investigation of the effects of standing desk use on reading comprehension and creativity. In a counterbalanced, within-subjects design, 96 participants completed reading comprehension and creativity tasks while both sitting and standing. Participants self-reported their mood during the tasks and also responded to measures of expended effort and task difficulty. In addition, participants indicated whether they expected that they would perform better on work-relevant tasks while sitting or standing. Despite participants’ beliefs that they would perform worse on most tasks while standing, body position did not affect reading comprehension or creativity performance, nor did it affect perceptions of effort or difficulty. Mood was also unaffected by position, with a few exceptions: Participants exhibited greater task engagement (i.e., interest, enthusiasm, and alertness) and less comfort while standing rather than sitting. In sum, performance and psychological experience as related to task completion were nearly entirely uninfluenced by acute (~30-min) standing desk use.

## 1. Introduction

Recent studies indicate that employees spend about 62% of the workday sitting [1], and a majority of university students (95.3%) report sitting down for 75% or more of their class time [2]. Occupational sitting time is a well-established public health concern, as it is strongly linked with chronic disease and mortality in a growing number of prospective studies [3]. Furthermore, greater sitting time is closely tied to psychological health outcomes, such as depressive symptoms and anxiety [4]. To reduce sitting time, and in turn, improve health, some individuals have begun to implement standing desks or sit-stand workstations—that is, workstations that can adjust to either a seated or standing position. Indeed, several studies have found that the long-term use of sit-stand workstations produces cardio-metabolic health benefits, such as improved glucose levels at 1 [5] and 12 months [6]; reduced total cholesterol at 8 weeks [7]; increased healthy HDL cholesterol at 3 months [8]; and, improved cardio-metabolic risk scores at 12 months [6]. However, we note that this research is still in its nascent stages and further studies are needed. Finally, studies have also found that a single use of a standing desk significantly increases energy expenditure [9,10,11,12]. 

For standing desks to be feasible for occupational and academic use, they must not only promote health, but also pose minimal to no detrimental threat to work performance. Previous research has revealed that compared to sitting only, standing or sit-stand workstations do not significantly alter typing or computer mouse performance [9,13,14,15,16,17,18], accuracy in identifying and correcting misspelled words within text [19], speaking quality [10], X-ray baggage screening [16], call center workers’ objective performance [20], or aspects of cognitive functioning such as attention, information processing speed, or short-term memory [21].

Despite these areas of accumulating knowledge, we know of no prior studies examining the impact of standing desks on reading comprehension and creativity. Importantly, the vast majority of occupations seem to necessitate at least a moderate amount of reading comprehension abilities. According to one study, roughly 90% of all jobs require medium to high levels of reading comprehension [22]. In addition, creativity represents another important component of occupational success [23], and it is required in many fields of work [24]. Plucker and colleagues [23] define creativity as, “the interaction among aptitude, process, and environment by which an individual or group produces a perceptible product that is both novel and useful as defined within a social context.” There are a number of large industries in which novel products are in high demand and competition is fueled by a search for novelty [25]. Creative industries were first defined as “activities which have their origin in individual creativity, skill, and talent and which have the potential for wealth and job creation through generation and exploitation of intellectual property” [26]. Examples include the advertising, software and computer services, research and development, publishing, architecture, engineering, and film industries [24]. Although many individuals think of creativity as an innate personality trait [27], decades of research have dispelled this myth, instead showing that creativity can in fact be fostered by environmental techniques [23]. 

In addition to assessing the effects of standing desks on task performance, it is important to examine potential psychological impacts, as workstations that have detrimental psychological effects would likely not be feasible for continued use. Only a few previous studies have evaluated the impact of standing desk use on perceived task effort [10] and workload [16], finding no effects on either outcome after acute use [28]. Furthermore, it is critical to assess individuals’ expectations about how standing desks will impact performance, as negative expectations may be an obstacle preventing individuals from utilizing standing desks and realizing their benefits. Research on this topic is limited and inconsistent; in one study employees expressed concerns that intervention strategies to reduce occupational sitting would harm employee focus and productivity [29], whereas in another study only 8% of college students predicted that standing desks would decrease academic performance [2]. Finally, although previous studies have evaluated the impact of standing desks on discomfort [14,17], fatigue [30], and alertness [17], we know of no acute studies to date that have assessed a broader range of self-reported emotions that may plausibly be experienced while completing work tasks (e.g., interest, enthusiasm, stress, distraction). Only one chronic study has examined a larger number of psychological states in relation to standing desks, finding that 4-weeks of use improved employees’ mood states, including reduced feelings of tension, confusion, depression, and total mood disturbance [28].

To address the aforementioned gaps in the literature, we conducted the first known experiment to test for differences in reading comprehension and creativity performance as a function of standing versus sitting at a desk. Given that we assessed the effects of a short-term, single use of a standing desk, we based our hypotheses primarily on prior studies in related domains that were also acute in duration [9,10,13,16,17,19]. First, we hypothesized that reading comprehension, creativity, and perceived difficulty and effort would not differ between seated and standing positions. In addition, we predicted that individuals would report experiencing higher levels of positive emotions (and decreased levels of negative emotions) while working in the standing position [28]. Finally, we assessed whether individuals typically expected that they would perform better on work-related tasks while sitting or standing at a desk, and whether those expectations would map on to actual performance. Like other acute performance studies, we utilized a student sample [14,17] in a laboratory setting [9,10,13,14,17,19], rather than intervene in a sample of office workers in their usual work environments [15,16]. The reasons for this approach were twofold: (1) compared to office workers [31], university students spend just as large of a percentage of their required work hours sitting—if not more; in a study of nearly 1000 college students, 82.7% of students spent 100% of their class time sitting [2]; and (2) given that this was the first empirical test of the performance outcomes assessed here, we valued a greater degree of experimental control and internal validity, viewing external validity as a next step for future research to further examine any observed effects in additional populations and settings.

## 2. Materials and Methods

### 2.1. Pre-Screening

Participants were recruited from a university psychology subject pool for undergraduate students, and course research credit was granted for participation. Individuals interested in participating completed a brief online pre-screening survey (see Measures below). Eligible participants were aged 18 or older, did not report having any current major illness or injury, and reported fluency in the English language. A total of 96 participants were enrolled in the study, and completed the laboratory session as described below.

### 2.2. Study Design, Counterbalancing, and Random Assignment

The university Human Subjects Institutional Review Board approved all study activities. In this within-subjects design, all participants completed the same four test sections: two different reading comprehension sections (RC–A and RC–B), and two different creativity sections (CR–A and CR–B). The composition of these test sections is described further in the Measures, below.

The following is a basic overview of the lab day testing procedure. Before beginning any test sections, participants were instructed to assume their randomly assigned starting body position: sitting or standing. In their respective starting position, participants then completed their first two test sections, which always comprised one reading comprehension section (RC–A or RC–B) and one creativity section (CR–A or CR–B). Then, participants transitioned to their alternate, ending position. In their appropriate ending position, participants then completed their third and fourth test sections, which also always comprised one reading comprehension section (RC–A or RC–B) and one creativity section (CR–A or CR–B).

We executed a full counterbalancing scheme to control for possible order effects related to test section or body position. The order in which participants performed the four test sections was counterbalanced between-subjects. To achieve this, we created a list of 32 possible conditions (see Table 1). Conditions comprised two components: (1) the order of the starting and ending body positions (sitting or standing); and (2) one of the 16 unique testing orders in which the two reading comprehension tests and the two creativity tests could be taken. The 16 possible test orders were generated with the stipulation that the first two sections had to always include 1 reading comprehension and 1 creativity test; likewise, the final two sections had to always include 1 reading comprehension and 1 creativity section. This ensured that all participants would complete one reading comprehension and one creativity section using each body position.

Next, we compiled a spreadsheet wherein each of the 32 conditions appeared three times (to account for our projected sample size). The order of the conditions was then randomized. As each new participant enrolled in the study, s/he was assigned to the condition appearing next on the randomized condition list. A total of three participants completed each condition, creating a final sample size of 96 participants.

### 2.3. Study Cover Story

The study was described to participants as two separate research projects combined into one laboratory session: “The Markers of Health Study,” and “The Creativity, Cognition, and Behavior Study.” Participants read that, “One project aims to understand how posture (sitting and standing) relates to physiology (respiratory rate), and the other project seeks to better understand creativity and cognitive functioning in adults.” During the informed consent process, the experimenter told participants, “I’ll ask you to sit for part of the study and stand for part of the study while we take some physiological measurements. In order to get an accurate and reliable measurement, we need to take 30 min of physiological readings while you are in each posture. We want to make sure we make use of your time while we’re collecting the readings, so while they are being taken, we’ll have you complete some cognitive tasks for the second study for another lab’s research team.”

### 2.4. Workstation

The sit-stand workstation consisted of a 48″ × 30″ electronically adjustable standing desk with a digital display and memory preset (Jarvis, Xinchang, China). For the seated test sections, an office chair with back support, a foot rest, wheels, and no arm rests was used (#B1690-CS, Boss Office Products, Commerce, CA, USA). In addition, a 24″ × 36″ × ¾″ commercial-grade, anti-fatigue comfort mat (CumulusPRO, Busan, Korea) was placed underneath the chair during seated test sections and also used to stand on during standing test sections.

### 2.5. Procedure

After participants provided informed consent, the experimenter attached a non-invasive elastic respiration band around the torso. Then, the experimenter left the room for 3 min to simply give participants time to get used to the sensation of the respiration band on the body. During this time, participants completed filler items designed to support the cover story that one of the studies was concerned with markers of health (e.g., typical hours of sleep per night, smoking history, and presence of any current diagnosed psychiatric or cognitive condition).

Next, participants assumed their randomly assigned starting body position according to their condition. Participants assigned to be seated for their first two test sections remained seated; those assigned to begin standing were asked to stand. When standing positions were assumed, the experimenter raised the desk height to the number of inches estimated to be appropriate for each particular participant, based on the height that they had reported in pre-screening. These recommended heights for standing desk use were acquired using an online calculator for ergonomic office desks (www.thehumansolution.com). After raising the standing desk, the experimenter showed participants where the desk adjustment controls were and encouraged them to alter the desk height to whatever level felt most comfortable to them. Participants assigned to be sitting for their first two test sections were also oriented to the desk adjustment controls and encouraged to alter the desk height to a comfortable level if needed.

After participants had assumed their starting body position, they then completed their first reading comprehension and creativity test sections. Next, participants self-reported how difficult they perceived each of the sections to be, how much effort they put into each section, and their mood during each section (see Measure*s* below). Then, participants switched to their respective ending (alternate) body position; there was no break or period of rest between the two positions. After completing their remaining test sections in their ending position, participants again completed measures of perceived difficulty, effort, and mood—this time in reference to their most recently completed reading comprehension and creativity test section experiences.

Participants then completed surveys assessing their physical activity in the past week, as well as their expectations about whether they would perform better on work-related tasks while sitting or standing at a desk. The experimenter then removed the respiration band, measured participants’ weight and height, completed a study debriefing, and granted participants their course credit.

### 2.6. Measures

#### 2.6.1. Demographic Characteristics

In the pre-screening survey, participants reported their age, sex, race/ethnicity, height, weight, whether they had a current major illness or injury, whether English was their primary language, and whether they had ever “regularly used a standing desk to perform their work/school duties without an office chair.” We entered the final item in exploratory analyses to test whether prior standing desk use would moderate any effects of body position on task performance.

#### 2.6.2. Reading Comprehension

Reading comprehension was assessed using materials from the Graduate Record Examination General Test (GRE)—a standardized, graduate-level admissions exam, typically taken by individuals who are interested in pursuing a masters degree, business degree, or doctoral degree [32]. At thousands of graduate and business schools [32], admissions and fellowship panels review applicants’ GRE scores as a qualification to guide their decision-making processes. From July 2015 to June 2016, a total of 584,677 people took the exam in over 194 countries, with non-U.S. citizens making up 43% of test takers [33]. The GRE is composed of verbal reasoning, quantitative reasoning, and analytic writing measures. Meta-analytic research examining data from 1753 independent samples has found the GRE—including the verbal reasoning section in particular, which contains the reading comprehension items—to be a valid predictor of outcomes such as graduate grade point average, degree attainment, publication citation counts, and faculty ratings [34].

The specific GRE materials administered in the present study were obtained from the official GRE practice exam, provided by Educational Testing Services online (www.ets.org/gre). In the present study, participants completed only the reading comprehension items from the each of the two verbal reasoning sections of the official practice exam. Previous studies assessing reading comprehension have also used GRE reading comprehension items from a sample exam [35,36]. The official GRE test makers state that the purpose of the verbal reasoning measure is to assess the “ability to analyze and draw conclusions from discourse, understand multiple levels of meaning, select important points, and understand the meanings of sentences and entire texts” [32]. 

The two administered reading comprehension test sections (RC–A and RC–B) each contained six passages (five 1-paragraph passages and one 2-paragraph passage) and multiple-choice questions related to those passages. The 1-paragraph passages contained a range of 65 to 202 words each, and the 2-paragraph passages contained 202 words (RC–A) and 463 words (RC–B), respectively. RC–A contained 12 questions, and RC–B contained 13 questions. Participants were given a total of 20 min to complete each section. Participants were instructed to let the experimenter know if they finished before the 20 min had elapsed; if this occurred, participants were then given the instructions for the next part of the study. The experimenter recorded the total amount of time that each participant spent completing each reading comprehension section. Performance on each section was calculated as the proportion of questions answered correctly.

#### 2.6.3. Creativity

We assessed creativity using the Wallach and Kogan Creativity Test [37]. Participants were asked to generate original uses for common objects (i.e., bricks and knives), instances of common concepts (i.e., things that are loud and round), and consequences of hypothetical events (i.e., what would happen if people went blind or no longer needed to sleep). CR–A comprised the “knife,” “loud,” and “blind” tasks; CR–B comprised the “brick,” “round,” and “sleep” tasks. Participants were specifically instructed to “write down all of the unusual, creative, and uncommon” responses that they could think of, as previous research has shown that giving instructions to be creative improves the validity of divergent thinking scores [38,39]. The experimenter read the instructions aloud to participants, and instructions were also given in print. Participants were given a total of 3 min to complete each task. This amount of time was controlled and invariable. At the end of each task, participants were instructed to “take a moment to evaluate your responses, and draw a circle around the two responses that you think are your most creative ideas”.

Three independent raters rated each response to each of the six tasks on a scale ranging from 1 (not at all creative) to 5 (highly creative). The raters were undergraduate research assistants who did not have any prior experience with scoring the Wallach and Kogan Creativity Test [37]. All raters completed a training session with the primary investigator (Laura Finch) to review the published scoring instructions [39] and complete practice ratings prior to assigning study response ratings. Ratings were given in accordance with the instructions provided by Silvia et al. [39], which were in turn adopted from Wilson, Guilford, and Christensen [40]. This method considers responses that are uncommon, remote, and/or clever to be creative. Raters considered all three of these qualities while completing their scoring, and allowed strength in one quality to balance weakness in another quality [39]. Raters also adhered to the following techniques endorsed by Silvia et al. (2008) and Harrington, Block, and Block [38]: (1) for the unusual uses tasks, raters gave lower scores for actual uses of the items (e.g., slicing something using a knife); and, (2) for the instances tasks, raters gave lower scores for objects that were present in the testing room that participants were in (e.g., paper towels or chair wheels as instances of things that are round). 

Across the six creativity tasks, participants generated a total of 4976 responses. Responses were handled and scored following the methodology of Silvia et al. [39]. Participants’ written responses were typed into a spreadsheet and sorted alphabetically within each task, which importantly served to blind raters to factors such as participant handwriting, the total number of responses each participant gave for each task, the position of each response within a participant’s set, and whether participants circled a response as one their top two best. Before beginning to score responses for a given task, the raters read all responses for that task to form an impression of the range in responses. Inter-rater reliability across the three independent raters was found to be acceptable (intraclass correlation coefficient = 0.73). An average rating was computed for each individual response within each task by calculating the mean across the three raters’ scores.

We then computed two creativity indices found by Silvia et al. [39] to be valid and reliable: average scoring and Top 2 scoring. To calculate average creativity, first, each participant’s average individual response ratings for a given task were summed and then divided by their total number of responses for that task. In this way, this average creativity index represents the overall creativity of a participant’s responses for a task and penalizes participants for generating a greater number of uncreative responses. Then, for each creativity section (sitting and standing), the average ratings for each of the three tasks within that section were also averaged, creating a final overall average creativity score for each body position to be used in statistical analyses.

In contrast, for the Top 2 creativity index, an average was calculated for each task for the two responses that participants indicated were their best two. Thus, this Top 2 creativity index was not affected by the total number of responses that participants generated for each task (note: for each task, over 96% of participants generated three or more responses). To compute this index, for each creativity section (sitting and standing), the Top 2 creativity averages for each of the three tasks within that section were also averaged, creating a final overall Top 2 creativity score for each body position to be used in hypothesis testing.

#### 2.6.4. Perceived Task Difficulty, Effort, and Mood

Participants were instructed to think back to their experience while completing each test section, and then were asked to rate how difficult it was (1 = not at all; 7 = extremely), as well as how much effort they put into it (1 = none; 7 = a lot). They were also asked to indicate the extent to which they experienced each of a number of emotions while completing each section (1 = not at all; 7 = extremely). Here, we modified the Positive and Negative Affect Schedule [41] to include an abbreviated list of 16 emotions. Given that this mood survey was to be completed a total of four times throughout the laboratory session (once for each test section), to minimize participant fatigue, we omitted emotions (guilty, scared, strong, hostile, proud, irritable, ashamed, and afraid) from the original 20-item measure that we did not hypothesize as being particularly conceptually relevant to completing work tasks or sitting/standing, and we also omitted emotions (excited, attentive, and active) that were somewhat conceptually related to other emotions already being assessed (interested, enthusiastic, alert). In addition, we added the items “stressed,” “tired,” “comfortable,” “distracted,” and “focused,” which we expected to be more relevant to the present study’s work-related tasks. Other prior standing desk studies have also assessed related constructs such as discomfort [14,17], fatigue, tension, and confusion [17].

#### 2.6.5. Performance Expectations

We created four items to assess participants’ expectations about how desk position would impact their performance on various work-related tasks. The prompt began, “Imagine that you are about to perform certain work-related tasks and you have the option of either sitting or standing at a desk.” Then, participants selected either “sitting” or “standing” in response to each of the four items. For example, participants were asked, “Do you think you would be more creative while sitting at a desk or while standing at a desk?” They also selected the position in which they believed that they would perform better on reading comprehension and typing tasks, as well as whether they thought they would “be a more productive writer” while sitting or standing at a desk.

#### 2.6.6. Physical Activity and Sedentary Behavior

The short form version of the International Physical Activity Questionnaire (IPAQ) [42] was used to assess participants’ usual general level of physical activity and sedentary behavior. This 7-item survey measures how much time participants spent doing various types of physical activity in the past 7 days, as well as how much time they spent sitting down (i.e., sedentary behavior). The following is a sample item: “During the last 7 days, on how many days did you do vigorous physical activities like heavy lifting, digging, aerobics, or fast bicycling?” Following the standard IPAQ scoring protocol [43], we computed total metabolic equivalent (MET) minutes per week and classified participants into one of three physical activity categories (low, moderate, or high). Given that there are no well-established thresholds for creating categories for sitting time [43], we used tertiles to categorize participants as exhibiting low, moderate, or high sitting time [44]; the resulting variable was used as a measure of sedentary behavior.

#### 2.6.7. Body Mass Index

The experimenter measured participants’ weight and height without shoes. Weight was assessed using a Tanita Professional Body Composition Monitor SC-331S, and height was measured using a stadiometer and recorded to the nearest 1/8 inch. Body Mass Index (BMI) was calculated using the standard formula (weight in pounds/height in inches^2^ × 703), and the standard BMI ranges were used to characterize each participant’s BMI category (underweight: <18.5; normal: 18.5 to 24.99; overweight: 25 to 29.99; obese: 30 or greater).

### 2.7. Statistical Analyses

As our primary analyses, we conducted paired *t*-tests to assess any effects of body position on the following dependent variables: GRE proportion correct, average creativity, Top 2 creativity, perceived difficulty of each task, perceived effort expended on each task, and emotions. Thus, we compared sitting and standing values for each of these within-subjects variables. In addition, we compared the average number of minutes that participants spent completing their seated and standing reading comprehension sections, as a significant difference between the two positions could potentially affect the study outcomes.

In exploratory analyses, to test whether body position effects on task performance may be moderated by prior standing desk experience, sedentary behavior, physical activity, or BMI, we added each of these categorical variables separately to repeated measures analysis of variance (ANOVA) models and examined the appropriate interaction terms involving body position.

In addition to assessing participants’ expectations about body position and task performance, we went on to test whether these expectations were indeed associated with actual performance differences between the two positions. We conducted point-biserial correlation tests to examine these relationships, entering expectations as a dichotomous variable and actual performance difference scores as a continuous variable. Expectations of enhanced performance while standing were coded as 1, and expectations of enhanced performance while sitting were coded as 0. We computed the difference in actual performance by body position for each outcome by subtracting sitting scores from standing scores.

SPSS software (version 24.0, IBM, Armonk, NY, USA) was used for all statistical testing, and the computer code is available. The level of significance for all tests was set at *p* < 0.05.

## 3. Results

### 3.1. Participant Demographics

Descriptive characteristics of the 96 study participants are presented in Table 2. We also note that among the 261 individuals who completed the study pre-screening, 8.4% (*n* = 22) self-reported prior regular experience with standing desks—a similar percentage to the laboratory sample.

### 3.2. Primary Analyses

As shown in Table 3, findings revealed no significant differences in performance, perceived task difficulty, or perceived task effort as a function of standing versus sitting. There was no difference in the number of minutes that participants took to complete their seated and standing reading comprehension sections, (*M* = 15.83, *SD* = 3.08 vs. *M* = 15.45, *SD* = 2.96, respectively; *p* = 0.18). The average total amount of time that participants spent completing the tests in each position was ~30–31 min (i.e., ~15–16 min for reading comprehension, and ~15 min for creativity (3 min for each of the 3 tasks; 1 min for the experimenter to read the instructions for each of the 3 tasks; and 1 min for participants to review their responses and circle their best two for each of the 3 tasks)).

Regarding mood, participants reported feeling more interested (*M* = 3.09, *SD* = 1.37 vs. *M* = 2.81, *SD* = 1.40, *p* = 0.008), enthusiastic (*M* = 2.05, *SD* = 1.19 vs. *M* = 1.90, *SD* = 1.18, *p* = 0.025), and alert (*M* = 3.66, *SD* = 1.47 vs. *M* = 3.40, *SD* = 1.58, *p* = 0.044) for the reading comprehension section that they stood for versus sat for. However, participants reported feeling more comfortable while sitting rather than standing for both the reading comprehension (*M* = 3.88, *SD* = 1.40 vs. *M* = 3.41, *SD* = 1.34, *p* = 0.001) and creativity tests (*M* = 4.25, *SD* = 1.33 vs. *M* = 3.83, *SD* = 1.33, *p* = 0.002). No other body position effects on mood were found for the reading comprehension or creativity tests for the remaining emotions: focused, inspired, motivated, determined, stressed, anxious, nervous, tired, jittery, distracted, distressed, and upset.

### 3.3. Moderation Analyses

As shown in Table 4, body position did not interact with the prior standing desk use, physical activity category, sedentary behavior category, BMI, or prior regular standing desk use to predict any of the three performance outcomes.

## 4. Discussion

Study findings revealed that standing at a desk did not impair (or enhance) performance on reading comprehension or creativity tasks relative to sitting at a desk, regardless of participants’ level of regular physical activity, sedentary behavior, BMI, or prior experience with standing desks. Although this is the first study to test experimentally how standing versus sitting affects these performance outcomes, these findings converge with other previous work demonstrating that standing desks do not hinder performance on a range of other work-related tasks [18]. Further evidence suggests that the use of sit-stand workstations either does not influence worker productivity, or may even enhance it. In their review of eight prior studies evaluating sit-stand workstations and work productivity, Karakolis and Callaghan [45] reported that four studies found no effect on productivity, three reported improved productivity, and one found mixed results. 

Additionally, in the present study, body position did not influence participants’ perceptions of how difficult the tasks were, nor did it affect the amount of effort that participants reported putting into the tasks. Previous research has similarly found that compared to sitting, acute standing desk use is associated with neither increased perceived effort related to a speech task [10], nor increased perceived workload related to a security screening task [16]. However, in another study, participants reported a significantly higher perceived workload when they stood versus sat while completing tasks [46]. Therefore, perhaps workload effects may depend on the nature of the task at hand, the duration of the relevant task, and/or the method of workload assessment.

When reflecting back on their mood during the tasks, participants reported feeling a similar level of most of the assessed emotions. However, there were a few exceptions for which statistically significant differences emerged: Participants reported feeling more interested, enthusiastic, and alert for the reading comprehension section that they stood for. However, we note that the difference in means for these emotions was relatively small (e.g., a difference of 0.28 on a 7-point scale for the interest item). Other studies that have acutely manipulated body position have found that, compared to sitting only, using a standing or sit-stand desk did not impact alertness [17] but reduced feelings of fatigue [30]. In addition, in the present study, participants felt less comfortable while standing for both types of tasks. Previous acute studies have been inconsistent regarding whether acute use of a sit-stand workstation increases [17] or decreases [14] musculoskeletal discomfort when compared to a traditional seated desk.

With regard to expectations, participants believed that they would be more creative while standing but believed that they would perform better while sitting in terms of reading comprehension, typing, and productivity of writing. These results differ from that of another study [2], wherein only 4–8% of individuals expected that standing desk use would impair performance. One reason for this discrepancy may be that the present study examined expectations about particular work tasks, whereas Benzo, Gremaud, Jerome, and Carr [2] investigated general performance. Another plausible explanation is that our study presented participants with only the forced choice of indicating whether performance outcomes would be better for standing or for sitting. If participants expected that performance would be no different between the two body positions, there was no means of expressing this opinion on the relevant survey. 

### 4.1. Study Implications

Our findings suggest that adopting a short-term standing position will not impair performance on reading comprehension or creativity tasks. In addition, standing at a desk seems to have minimal effects on acute emotional states, with the exceptions of enhanced interest, enthusiasm, and alertness during task completion. These findings converge with prior survey data indicating that most college students and instructors expect that standing desk use will improve engagement and attention [2]. The three observed mood enhancements appear to hang together to embody the construct of work engagement, defined as a “work-related state of mind that is characterized by vigor, dedication, and absorption” (p. 74) and also characterized by high energy and enthusiasm [47]. Enhanced work engagement has been strongly linked with well-being and performance [48]. Although taking a standing position improved engagement but not performance in the present study, enhanced engagement is valuable in its own right as a promoter of well-being. Given that some individuals associate prolonged sitting with de-motivation [29], future studies should examine whether spreading knowledge about standing desks and improved engagement may therefore promote the use of these workstations. 

Study results also suggest that individuals may hold unfounded expectations about how standing desks will affect future performance on work-related tasks. Participants believed that compared to a seated position, standing would be more beneficial for creativity but worse in terms of reading comprehension, typing, and productivity of writing. The former two beliefs were not supported by results from this study, and moreover, participants’ expectations of differential performance between seated and standing positions were not associated with any actual performance differences between these positions. Furthermore, previous research has shown that typing performance is also typically unaffected by standing desk use [9,13,16,19]. Therefore, an overall theme seems to be emerging, in which individuals’ (mostly negative) standing desk performance expectations are not supported by any strong empirical evidence. We recommend that the growing scientific data refuting these expectations be better disseminated to students with the aim of combatting this potential obstacle to standing desk use. 

Our study findings suggest that if colleges and universities should incorporate sit-stand workstations on their campuses with the aim of promoting student health, the short-term use of these workstations may not incur detriments to students’ task performance and psychological well-being. As an alternative to standing desks, some individuals have been implementing treadmill desks in their work environments to promote health. Like standing desks, treadmill desks have also demonstrated positive effects for physiological functioning [18] and do not seem to harm reading comprehension performance [35,49]. There is also some evidence that treadmill desks may improve creativity among college students, relative to sitting [50]. However, compared to standing desks, treadmill desks also carry the disadvantages of a far greater monetary cost, as well as decreased fine motor skills and math problem solving among students [35]—factors that point to standing desks as a more feasible option for this population.

### 4.2. Strengths, Limitations, and Future Directions

This study was strengthened by its within-subjects, fully counterbalanced, randomized design. Furthermore, a recent review [18] identified eight prior studies investigating standing desk use and work performance, and these studies included a range of 12–60 participants each, with a mean of approximately 31 participants per study. In contrast, the present study included a sample over three times as large, providing increased statistical power. Moreover, to our knowledge, none of these prior studies made use of a study cover story, and thus, the researchers’ true study aims were likely extremely transparent to participants, potentially eliciting biased responses. In contrast, our study addressed and minimized this concern by including a study cover story. 

An additional strength of the study was its inclusion of a relatively diverse sample, with approximately 70% of participants identifying as belonging to a racial/ethnic minority group. It is especially important to examine the impact of standing desks in minority populations, as some minority groups are disproportionally vulnerable to the types of negative health conditions associated with greater sitting time [51], and thus, the feasible utilization of standing desks among these populations may be a potential avenue for mitigating racial/ethnic health disparities. 

Despite these strengths, no study is without limitations. First, we may not yet infer the generalizability of the present study’s findings to populations that were underrepresented in the present study. The majority of participants were young adult university students; thus, future research should examine the present outcomes in a sample of employees from a broader age range and work experience history, ideally in their current regular workplace setting. Nonetheless, the tasks that participants completed were applicable to most work settings, and conducting the study in this student population represented a feasible, highly internally valid initial investigation into these novel performance outcomes. In addition, we observed limited variability in several of the moderator variables, such that the study sample primarily consisted of individuals who were very physically active and had never used a standing desk before. Future studies should include a wider range of individuals, such as those who are less physically active or have had more previous experience with standing desks.

Another limitation of the study was its assessment of relatively short-term effects of standing desk use. However, several studies have examined the impact of sit-stand workstations on a longer time scale and similarly found no effects on other aspects of work performance. For example, 5 days of use did not impact data entry performance [14], and 4 weeks of use did not influence workers’ self-reported performance [5]. Another study similarly found no effects on self-reported work performance across a 3-month period, yet the sit-stand workstations significantly decreased sitting time by over 2 h/day [8]. Furthermore, 4 [52] to 8 weeks of use [7] effectively reduces sitting time without impacting productivity. In sum, using sit-stand workstations for a month or more appears to reduce sitting time without harming general work performance and productivity. 

## 5. Conclusions

This study presents the first evidence that compared to the use of traditional seated desks, the short-term use of a standing desk does not seem to impair reading comprehension or the ability to generate creative ideas. Although future studies should evaluate whether these areas of task performance remain unaffected by longer-term standing desk use in the workplace, these initial findings suggest that if university students choose to use standing desks in an effort to reduce sitting time or promote health, doing so may increase their short-term task engagement without undermining work performance. 

## Figures and Tables

**Table 1:** Counterbalancing of Body Position and Testing Orders.

Condition	Testing Order	Starting Position	First Section	Second Section	Ending Position	Third Section	Fourth Section
1	1	Sit	RC–A	CR–A	Stand	RC–B	CR–B
2	2	Sit	RC–A	CR–B	Stand	RC–B	CR–A
3	3	Sit	RC–B	CR–A	Stand	RC–A	CR–B
4	4	Sit	RC–B	CR–B	Stand	RC–A	CR–A
5	5	Sit	RC–A	CR–A	Stand	CR–B	RC–B
6	6	Sit	RC–A	CR–B	Stand	CR–A	RC–B
7	7	Sit	RC–B	CR–A	Stand	CR–B	RC–A
8	8	Sit	RC–B	CR–B	Stand	CR–A	RC–A
9	9	Sit	CR–A	RC–A	Stand	CR–B	RC–B
10	10	Sit	CR–A	RC–B	Stand	CR–B	RC–A
11	11	Sit	CR–B	RC–A	Stand	CR–A	RC–B
12	12	Sit	CR–B	RC–B	Stand	CR–A	RC–A
13	13	Sit	CR–A	RC–A	Stand	RC–B	CR–B
14	14	Sit	CR–A	RC–B	Stand	RC–A	CR–B
15	15	Sit	CR–B	RC–A	Stand	RC–B	CR–A
16	16	Sit	CR–B	RC–B	Stand	RC–A	CR–A
17	1	Stand	RC–A	CR–A	Sit	RC–B	CR–B
18	2	Stand	RC–A	CR–B	Sit	RC–B	CR–A
19	3	Stand	RC–B	CR–A	Sit	RC–A	CR–B
20	4	Stand	RC–B	CR–B	Sit	RC–A	CR–A
21	5	Stand	RC–A	CR–A	Sit	CR–B	RC–B
22	6	Stand	RC–A	CR–B	Sit	CR–A	RC–B
23	7	Stand	RC–B	CR–A	Sit	CR–B	RC–A
24	8	Stand	RC–B	CR–B	Sit	CR–A	RC–A
25	9	Stand	CR–A	RC–A	Sit	CR–B	RC–B
26	10	Stand	CR–A	RC–B	Sit	CR–B	RC–A
27	11	Stand	CR–B	RC–A	Sit	CR–A	RC–B
28	12	Stand	CR–B	RC–B	Sit	CR–A	RC–A
29	13	Stand	CR–A	RC–A	Sit	RC–B	CR–B
30	14	Stand	CR–A	RC–B	Sit	RC–A	CR–B
31	15	Stand	CR–B	RC–A	Sit	RC–B	CR–A
32	16	Stand	CR–B	RC–B	Sit	RC–A	CR–A

Note: RC = reading comprehension; CR = creativity. Each of the 32 conditions comprised both a body position order and a testing order. For example, participants in condition 32 first completed creativity test B, followed by reading comprehension test B (both while standing); then, these participants moved to a seated position and completed reading comprehension test A, followed by creativity test A.

**Table 2:** Participant Demographic Characteristics.

Variable	Response or Category	*n*	*M (SD)* or %	Min-Max or *Mdn* (IQR)
Age (years)		96	20.92 (3.02)	18–36
Sex	Female	77	80.2	
	Male	19	19.8	
Race/ethnicity	Asian/Pacific Islander	34	35.4	
	White	29	30.2	
	Hispanic/Latino	12	12.5	
	Bi-racial	11	11.5	
	Arabic/Middle Eastern	6	6.3	
	Black	3	3.1	
	South Asian	1	1.0	
Physical activity (MET-min/week)		96		4761 (4929)
Physical activity category	Low	1	1.0	
	Moderate	21	21.9	
	High	74	77.1	
Sitting time (hours/day) ^a^		92		7.00 (2.38)
Sedentary behavior category	Low	23	25.0	
	Moderate	32	34.8	
	High	27	40.2	
Body Mass Index (kg/m^2^)		96	23.23 (3.67)	17.20–34.16
Body Mass Index category	Underweight	5	5.2	
	Normal Weight	64	66.7	
	Overweight	21	21.9	
	Obese	6	6.3	
Prior regular standing desk use	No	88	91.7	
	Yes	8	8.3	

Note: IQR = interquartile range; MET = metabolic equivalent of task. ^a^ Following standard IPAQ scoring protocol [43], four extreme values of >16 h/day were omitted, as it is assumed that individuals spend an average of 8 h/day sleeping.

**Table 3:** Performance and Psychological Outcomes by Position.

Variable	Standing		Sitting		Statistic	
	*M*	*SD*	*M*	*SD*	*t*(95)	*p*
Reading comprehension						
Proportion correct	0.45	0.18	0.45	0.17	0.00	0.98
Perceived difficulty	4.44	1.11	4.59	1.01	0.91	0.09
Perceived effort	4.73	1.36	4.65	1.33	0.84	0.36
Creativity						
Average creativity	2.11	0.33	2.12	0.33	0.07	0.80
Top 2 creativity	2.41	0.48	2.41	0.39	0.00	0.98
Perceived difficulty	4.04	1.53	3.90	1.55	1.32	0.19
Perceived effort	5.08	1.30	5.17	1.25	0.88	0.35

Note: Reading comprehension proportion correct is on a scale from 0 to 1.0. Average creativity and Top 2 creativity ratings are on a scale from 1 (*not at all creative*) to 5 (*highly creative*). Participants rated how difficult each test was (1 = *not at all*; 7 = *extremely*), as well as how much effort they exerted on it (1 = *none*; 7 = *a lot*).

**Table 4:** Analyses Testing Moderation of Position-Performance Relationships.

Moderator		Reading Comprehension		Average Creativity		Top 2 Creativity	
	*df*	*F*	*p*	*F*	*p*	*F*	*p*
Physical activity category	2,93	0.14	0.87	1.78	0.18	1.43	0.25
Sedentary behavior category	2,89	0.72	0.49	0.23	0.80	0.45	0.64
Body Mass Index category	3,92	0.64	0.59	0.25	0.87	0.27	0.85
Prior regular standing desk use	1,95	0.004	0.95	0.20	0.66	0.24	0.63
